# Ethics for surgeons during the COVID-19 pandemic, review article

**DOI:** 10.1016/j.amsu.2020.06.003

**Published:** 2020-06-08

**Authors:** Denis W. Harkin

**Affiliations:** Royal Victoria Hospital, Belfast Health & Social Care Trust, Northern Ireland, UK

**Keywords:** Surgeons, Surgery, Ethics, Professionalism, Pandemic, Covid-19

## Abstract

The Covid-19 pandemic is a devastating global healthcare emergency with seismic impact on how modern surgical services function. Surgeons worry, that whilst healthcare-resources are directed against the pandemic, double effect may predict these benevolent public health efforts will cause unintended maleficent effects through delays to surgical treatment. Surgeons will make many challenging ethical judgements during this pandemic, here we conduct a narrative review of how medical ethics may help us make the best available choices.

A narrative review of all the relevant papers known to the author was conducted. We discuss the key aspects of medical ethics, and how they have applied to surgeons during the Covid-19 pandemic.

The four fundamental principles of medical ethics include: Beneficence, Nonmaleficence, Autonomy and Justice. Surgeons will face many decisions which shall challenge those ethical principles during the pandemic, and wisdom from medical ethics can guide surgeons, to do the right thing, make best available choices, and get the best available outcome for patients during the Covid-19 pandemic.

The practice of surgery is distinguished by good judgement in the face of uncertainty, we must strive to do the right thing, advocate for our patients, and be honest in the face of uncertainty. Medical Ethics can guide us to make the best available choices for our patients during the Covid-19 pandemic, afterwards, we must emerge wiser having learnt lessons and rebuilding trust in surgical care.

## Highlights

• The Covid-19 pandemic is a devastating global healthcare emergency with seismic impact on how modern surgical services function.
• Surgeons worry, that whilst healthcare-resources are directed against the pandemic, double effect may predict these benevolent public health efforts will cause unintended maleficent effects through delays to surgical treatment.
• Surgical practice is distinguished by good judgement in the face of uncertainty, we must strive to act ethically, be honest, do the right thing, and advocate for our patients.

## 1 Introduction

The Covid-19 pandemic [1,] is a devastating global healthcare emergency with seismic impact on how modern surgical services function. Surgeons worry that whilst our Healthcare efforts are correctly focused on increasing critical care capacity, infection control, and the multi-pronged strategies to defeat covid-19, the unintended harm caused by cancelled surgical treatment will become huge [3,4].

Severe Acute Respiratory Syndrome Coronavirus-2 (SARS-CoV-2) transmission has caused a global pandemic of Coronavirus Disease 2019 (COVID-19), from asymptomatic or mild illness to sudden hypoxemic respiratory failure, multisystem organ failure and death [1,2]. Globally, millions have been infected and hundreds-of-thousands have died, amongst them many frontline Healthcare Workers and Surgeons [1,2,5,6]. Epicentres, even in well-resourced healthcare systems, have been overwhelmed and have diverted all available resources to the pandemic surge [5,6]. Surgeons, along with their fellow Healthcare workers, have selflessly placed themselves at-risk to deliver emergency care.

Surgeons face unique specialty-specific challenges during this pandemic, including increased personal risk from intra-operative infection and the professional challenges of prioritisation of who receives the limited surgical care available. Surgeons’ are also conscious that significant collateral damage will arise from delays to urgent surgical treatment and from backlogs of postponed surgical procedures [3,4]. Surgical Societies have provided guidance by consensus for surgical prioritisation [4] but resultant secondary-harm caused will only fully emerge after the pandemic from multi-national research collaboratives, such as the Global Surgery “CovidSurg Modelling Studies” [7].

So how do we practice surgery ethically during this pandemic? The four fundamental principles of medical ethics as defined by Beauchamp and Childress [8], also considered the building-blocks of people's morality, include: Beneficence, Nonmaleficence, Autonomy and Justice. In practice, ethics will involve the interaction between surgeons and patients which should be conducted with fairness, honesty, compassion and respect. We present a narrative review of all the relevant papers known to the author and discuss the key aspects of medical ethics as they may apply to surgeons during the Covid-19 pandemic. We discuss how medical ethics may guide surgeons, to do the right thing and get the best available result for their patients during and after this Covid-19 pandemic.

## 2 Beneficence, nonmaleficence and best interests

Beneficence is to care for, or help, others and “do good”. Nonmaleficence is to “do no harm”. The ancient Oath of Hippocrates [9] bound a physician to act “for the benefit of my patients, and abstain from what is deleterious or mischievous”. The primacy of patient welfare is the foundation of Medical Ethics, and assurance of those values form the bedrock of most professional codes [10,11]. Doctors have a primary responsibility to act in a patient's best interests, without being influenced by any personal consideration, and patients must have trust in us to do the right thing. During this pandemics some individual best-interests must come secondary to that of society, for the greater good.

The patient-doctor relationship has always been a privileged one, where patients place their trust in their doctors to act in their best interests. That trust will be challenged during the pandemic as surgeons and surgical services cannot function as normal, especially if unintended harm to patients results. However, certain decisions are clearly beyond our control. During the pandemic most healthcare systems have stopped all but emergency surgical care, indeed in many areas a moratorium has been placed on scheduled surgery [12,13]. Even the remaining emergency surgical care is restricted, with diminished critical care support, and the need to balance increased risks from complex surgery with attendant risk of contraction of Covid-19 in the peri-operative period [13]. Surgical societies have provided support to front-line surgeons by established criteria by expert consensus for triage and prioritisation in order to identify procedures that can be postponed until after the pandemic and those that should not [3,4,12,13]. Worldwide most deaths have occurred in elderly patients with comorbid disease and we know operations on Covid-19 positive patients carries a high mortality, but we must not consider all surgery futile in older, infirm or Covid-19 positive patients [4]. Our duty is to protect the most vulnerable but Doctors are under no obligation to offer treatment they consider futile [14]. However, we cannot withhold care entirely from certain groups and risk an avoidable cull of the elderly and infirm during the pandemic, but rather we should apply an individualised and context-specific approach to risk assessment [15]. Indirect harm, will take many forms, including: lost curative cancer surgery; increased stomas and amputations from damage-control surgery; fatalities from delayed cardiac, vascular, or neurosurgical operations. Cancer patients are a particular vulnerable group, contracting Covid-19 during treatment exposes them to a higher mortality but delays in cancer surgery may also lose opportunity for cure [16]. Cancer Networks have reorganisation to reduce the direct and indirect impact on cancer mortality by providing neo-adjuvant therapy and some essential surgery through “clean” centres supported by telemedicine, Covid-19 Testing, Isolation-Protocols and even anti-viral pharmacotherapy [16]. The doctrine of double effect, where an action intended for good unintentionally causes harm, predicts how these benevolent public health initiatives can have maleficent effects. A balance must be struck between postponing treatment that is currently too risky, and continuing to save the lives of patients with urgent health needs unrelated to covid-19. Surgical leaders must remain vigilant and when local circumstances permit advocate for cautious and safe staged re-introduction of surgical services prioritised by clinical need and working across specialties to clear the backlog [4,[17], [18], [19]].

## 3 Altruism and duty

Covid-19 Hospital-based transmission has occurred [2,5,6] and Surgeons face particular risks due to intimate physical proximity and contact with potentially infectious bodily fluids, blood, urine and faeces. Sadly, Surgeons have been exposed unknowingly to large viral-loads early in the pandemic, especially amongst Ophthalmic, Oto-laryngology, Maxillo-Facial and Thoracic Surgeons. Other Surgeons have become ill or died in the course of delivering emergency surgical care or re-deployed to support overstretched critical care services. Altruism is the selfless concern for the well-being of others, and Surgeons will selflessly place themselves at risk to help patients and support colleagues. Surgeons have willingly redeployed to assist other front-line services in critical care and emergency departments. Teams have worked flexibly to cover vacant roles and maintain emergency surgical care [18,19]. Patients have still benefitted from urgent or emergency operative intervention for time-sensitive disease processes such as malignant neoplasia or for true emergencies such as perforated viscus, bleeding, ischaemia or traumatic injury [3,4,[17], [18], [19]]. To manage the risk of Covid-19 transmission persons presenting for surgical intervention are suspected of infection (and thus transmissibility) even if asymptomatic and treated accordingly [18,19]. Surgeons have demonstrated that it is possible to provide safe surgical care even for SARS-CoV-2-positive patients, whilst minimizing nosocomial transmission to healthcare workers [13,18,19]. However, it is vital that infection prevention and control measures are robust, patients risk stratified by COVID-19 testing and staff protected with personal-protective equipment (PPE) and environmental shielding, otherwise isolated or sick staff will further deplete surgical care. Early in the Pandemic Surgeons identified as their key priorities, in the following order: the need to maintain emergency surgical capabilities, to protect and preserve the surgical workforce, and fulfil alternate surgical roles within the team or non-surgical roles on redeployment [3]. Surgeons have demonstrated altruism, done their duty, and worked collaboratively to share surgical experience and striven to provide non-surgical care competently with upskilling and support from colleagues.

## 4 Autonomy and informed consent

Autonomy, is to respect another's wishes. Surgeon-patient relationship should be considered a partnership, in which the surgeon's duty is to honestly educate and empower patients to make appropriate informed choices about surgical care [11]. People have the right to control what happens to their bodies including the refusal of treatment, because they are free and rational, and these decisions must be respected by everyone, even if those decisions aren't in the best interest of the patient. In law, the principle of autonomy is often taken to bestow a negative right, a right to non‐interference [8,11,14]. To interpret autonomy positively, by contrast, would arguably entitle everyone to any requested treatment, regardless of medical advisability or competing claims for scarce resources. A positive interpretation of autonomy is therefore often taken to be incompatible with the ethical principles of non‐maleficence (do no harm), justice (distribute scarce resources fairly) and with the practical realities of healthcare provision especially during a global pandemic [8,11,14]. The combined effects of a moratorium on elective surgery and annexation of private surgical facilities have meant patient choice has been restricted. More concerningly in epicentres where healthcare systems have been overwhelmed by the pandemic surge finite resources such as critical care beds and ventilators have not been available for all who may have benefitted and patient choice has been removed [2,5,6,14]. In a pandemic some choices must be restricted or even withheld. Informed consent is ethically and legally required prior to invasive surgical procedures and should include a discussion of the risks, benefits and alternatives [11,20,21]. To be valid consent the patient must have capacity, have understood the relevant information, consented voluntarily and communicate that decision. There are many challenges to informed consent, especially in vulnerable patients, including patient-centred barriers (such as age, education, language, illness and disability) and process-centred barriers (forms, information, communication and timing). Communication barriers are increased during this pandemic by personal-protective equipment (masks and visors), social-distancing, and isolation from family or best-friends. Surgeons have made efforts to overcome those barriers with innovative use of proven digital and audio-visual interventions [22]. In emergency situations surgeons will continue and strive to act in patients best-interests.

## 5 Justice and healthcare rationing

Justice, is to act or treat justly or fairly. We should try to be as fair as possible when offering treatments to patients and allocating scarce medical resources. You should be able to justify your actions in every situation [8,10,11]. During a pandemic the individual patient's best interests must become secondary to that of society as a whole. Social justice in Healthcare demands we consider the available resources and the needs of all patients while taking care of an individual patient. In epicentres, the highest death rates coincide with breakdown of local healthcare systems. Even well-resourced healthcare systems, overwhelmed by demand for life support and ventilators have had insufficient for all in need, and finite resource has had to be directed to those most likely to survive [9,10]. These grave decisions should not be taken in isolation but working in partnership and recognising the uncertainty that exists. In tackling the pandemic there are also grave risks of indirect harm to patients as diagnosis, treatment, procedures and surgeries are delayed. To honour the principle of beneficence, providers should try to relieve suffering to the best of their ability. In the aftermath a concerted effort must be made to provide redress for disadvantaged patients. Surgeons and Healthcare providers will need to work collaboratively and creatively to safely and sustainably restore surgical services cognisant of risks of a second pandemic surge and financially constrained by the pandemics economic devastation [4,18,19].

## 6 Confidentiality, conflicts and medical mistakes

Confidentiality is integral to patient-doctor trust [10,11]. With social-distancing during the pandemic we have witnessed a parallel outbreak of social-media usage and exploration of novel video-conferencing (VC) in healthcare. This process has been optimised by rapid upskilled of surgeons in best-practice in communications skills for telephone or audio-visual consultations [23]. Surgeons must temper their instinct to publicise experiences as they overcome adversity with novel approaches to protect the fundamental duty to protect patient confidentiality. Naturally, vital experience, evidence and research must be disseminated to ensure care is evidence-based [18,19] but must follow the principles of research ethics outlined in the Helsinki Declaration [24], ensure consent is informed, and declare and avoid conflicts of interest and by working collaboratively to be best, rather than first. Surgeons, like other citizens, must also endure social-distancing and a relative financial hardship with loss-of-earnings and for some who run small business and hire staff the real threat of insolvency. Indeed the economic effects of the Covid-19 pandemic on tariff-based healthcare systems such as the United States of America, where a moratorium had been placed on elective surgery, has been grave despite federal financial support measures. The ethical danger is that many providers may not survive unless a sustainable resumption of elective surgery can be achieved soon but financial needs must not take primacy over safety [25]. Whilst we endure these seismic events on surgical practice some medical mistakes will also happen, and can violate the principle of nonmaleficence, and here truthfulness and justice will guide us [26]. Learning how to prevent mistakes, openly reporting mistakes, and learning from mistakes helps us demonstrate respect and rebuild trust.

## 7 Conclusions

The practice of surgery is distinguished by good judgement in the face of uncertainty, we must strive to do the right thing, advocate for our patients, and be honest in the face of uncertainty. Medical Ethics can guide us to make the best available choices for our patients during the Covid-19 pandemic. We must emerge wiser having learnt lessons and rebuild trust in surgical care whilst respecting those principles of beneficence, nonmaleficence, autonomy and justice.

## Provenance and peer review

Not commissioned, externally peer-reviewed.

## Ethical approval

Not Applicable.

## Author contribution

I as Corresponding Author confirm the concept, design, data collection and analysis and interpretation, and writing are my own. I confirm there are no other contributors.

## Registration of research studies

Name of the registry: Not Applicable.

Unique Identifying number or registration ID:

Hyperlink to your specific registration (must be publicly accessible and will be checked):

## Guarantor

I the Contributing Author accept full responsibility for the work.

## Funding **source**

Not Applicable.

## Declaration of competing interest

I have read and understood the policy on declaration of interests and have no relevant interests to declare. The responsibility for the content lies with the author and the views stated herein should not be taken to represent those of any organisations or groups with and for which he works.
